# MicroRNA-301a-3p promotes pancreatic cancer progression via negative regulation of *SMAD4*

**DOI:** 10.18632/oncotarget.4124

**Published:** 2015-05-12

**Authors:** Xiang Xia, Kundong Zhang, Gang Cen, Tao Jiang, Jun Cao, Kejian Huang, Chen Huang, Qian Zhao, Zhengjun Qiu

**Affiliations:** ^1^ Department of General Surgery, Shanghai Jiaotong University Affiliated First People's Hospital, Shanghai, China; ^2^ Department of Pathophysiology, Key Laboratory of Cell Differentiation and Apoptosis and National Ministry of Education, Shanghai Jiaotong University School of Medicine, Shanghai, China

**Keywords:** miR-301a-3p, SMAD4, pancreatic ductal adenocarcinoma, xenograft tumor, prognosis

## Abstract

**Background:**

Aim to determine the clinicopathological and prognostic role of miR-301a-3p in pancreatic ductal adenocarcinoma(PDAC), to investigate the biological mechanism of miR-301a-3p *in vitro* and *in vivo*.

**Methods:**

By tissue microarray analysis, we studied miR-301a-3p expression in PDAC patients and its clinicopathological correlations as well as prognostic significance. qRT-PCR was used to test miR-301a-3p expression in PDAC tissues and cell lines. Functional experiments including *in vitro* and *in vivo* were performed.

**Results:**

Significantly higher expression of miR-301a-3p were found in PDAC patients with lymph node metastasis and advanced pathological stages and identified as an independent prognostic factor for worse survival. In PDAC samples and cell lines, miR-301a-3p was significantly up-regulated compared with matched non-tumor tissues and normal pancreatic ductal cells, respectively. Overexpression of miR-301a-3p enhanced PDAC cells colony, invasion and migration abilities *in vitro* as well as tumorigenicity *in vivo*. Furthermore, *SMAD4* was identified as a target gene of miR-301a-3p by cell as well as mice xenograft experiments. In PDAC tissue microarray, a significantly inverse correlation between miR-301a-3p ISH scores and *SMAD4* IHC scores were observed in both tumor and corresponding non-tumor tissues.

**Conclusion:**

MiR-301a-3p functions as a novel oncogene in PDAC and the oncogenic activity may involve its inhibition of the target gene *SMAD4*.

## INTRODUCTION

Pancreatic ductal adenocarcinoma(PDAC) is one of the most notorious cancers, with an overall 5-year survival rate of less than 5% and a median survival time of approximate 6 months [1]. Curative resection is the core of successful therapy to PDAC patients [2], but only 10%-15% of them are diagnosed at the early stages when surgical resection can be offered [3]. While over 80-90% subjects are diagnosed at the advanced stages [4]. Adjuvant chemotherapy has been shown to increase 5-year survival rate to 20%-30% despite conflicting evidences remains [5]. Accordingly, PDAC still refers to the lowest patient survival rate among any solid cancers [6]. Generally speaking, this dismal outcome is attributed to the aggressive local invasion, early metastasis and dissemination of the PDAC cells [7]. Hence, there is a desperate need in developing new diagnostic biomarkers as well as innovative therapeutic strategies to improve this situation.

MiRNAs are small non-coding RNA gene products of approximately 22 nucleotides that down-regulate gene expression by binding to the 3′untranslated regions(3′UTR) of specific target messenger RNAs (mRNAs), leading to mRNA degradation or inhibition of translation [8, 9]. It has been confirmed that miRNAs regulate many cellular processes such as cell growth, cycle, differentiation and apoptosis [10]. Amplification or overexpression of miRNAs even can down-regulate tumor suppressors or other genes involved in cell transforming, thereby contributing to tumor formation by stimulating proliferation, angiogenesis, and invasion; i.e., they act as oncogenes. Similarly, miRNAs could down-regulate different proteins with oncogenic activity; i.e., they act as tumor suppressors [11]. Furthermore, several clinical studies have observed correlations between miRNAs expression and clinicopathological characteristics or long-term survival [12]. The miR-301a-3p, which locates at chromosome 17q22, was shown to be up-regulated in many cancers, including breast cancer [13], gastric cancer [14], hepatocellular cancer [15]. However, the biological processes and molecular mechanisms underlying miR-301a-3p in PDAC remain poorly understood.

In this study, microarray-based strategy was applied to identify miR-301a-3p as a valuable parameter that correlated with clinicopathological features and prognosis. Meanwhile, we analyzed miR-301a-3p expression levels in PDAC tissues compared with matched non-tumor tissues and reported that miR-301a-3p promoted tumorigenesis in PDAC cell lines both *in vitro* and *in vivo*. We also identified *SMAD4* as one of the target genes of miR-301a-3p by cell and mice xenograft experiment. Our findings provided a potential oncogenic mechanism of miR-301a-3p orchestrates in PDAC.

## RESULTS

### Elevated miR-301a-3p expression correlated with lymph node metastasis, advanced pathological stage as well as serving as an independent prognostic factor of poor overall survival

**Figure 1:**
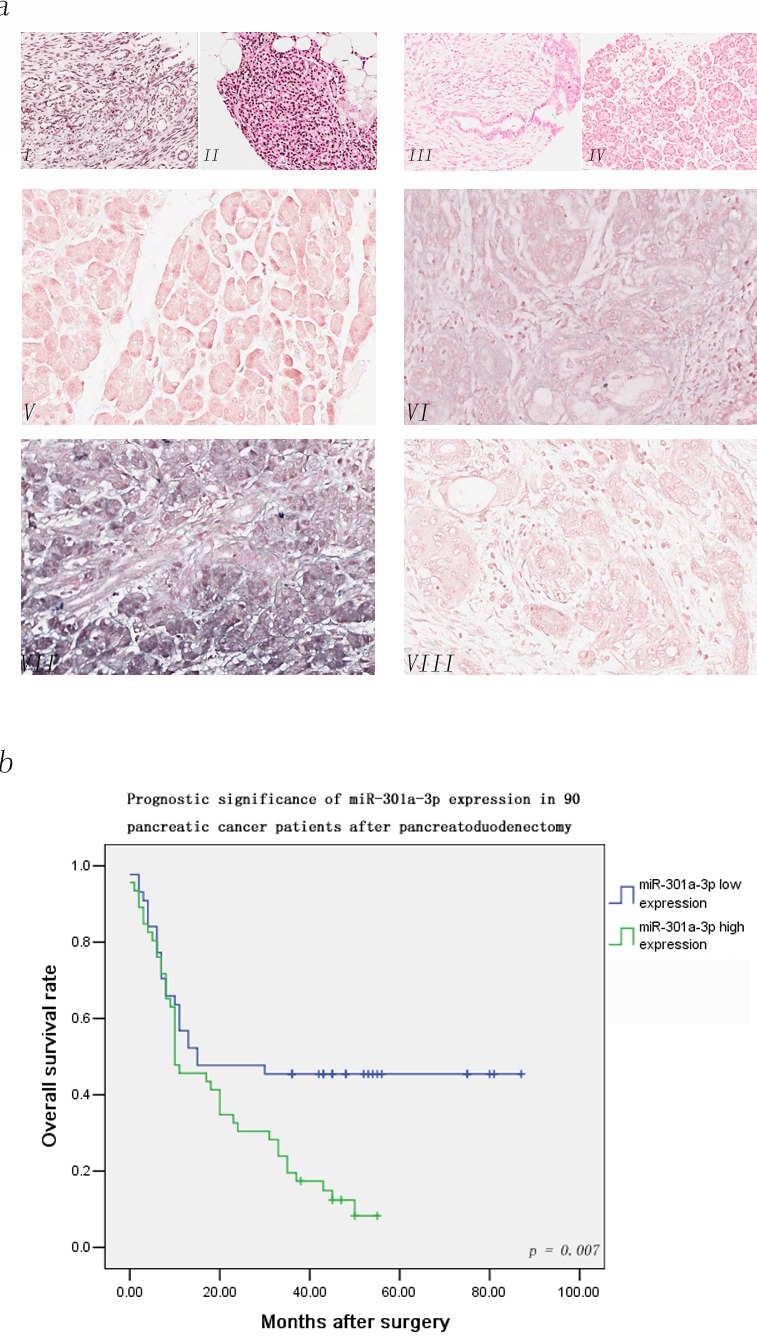
MiR-301a-3p detection in the tissue microarray of 90 PDAC patients by LNA-ISH *In situ* hybridization analyzes using 3′ and 5′ DIG-labled DNA probes complementary to miR-301a-3p were performed. The positive ISH staining was expressed as blue-violet. **a(I).** staining of U6 in cancer sample(positive control). **a(II)**. Staining of U6 in non-cancer sample. **a(III).** staining of scramble control probe in cancer sample(negative control). **a(IV).** Staining of scramble control in non-cancer sample. **a(V).** The expression of miR-301a-3p was negative in tumor tissue. **a(VI).** The weak expression of miR-301a-3p was detected in tumor tissue. **a(VII).** The strong expression of miR-301a-3p was detected in the tumor tissue. **a(VIII).** No signal was detected in the normal pancreatic tissue. **b.** The association between miR-301a-3p expression levels in tumors and overall survival in 90 PDAC patients using ISH score. Patients with high expression of miR-301a-3p display a significant shorter overall survival after surgery. Kaplan-Meier method and log-rank test were used to evaluate overall survival and compare the differences between the two groups, *p* = 0.007.

To elucidate the functional role of miR-301a-3p in PDAC, we utilized ISH to study the expression pattern of miR-301a-3p in tumors and corresponding non-tumor tissues. A significant high level ISH score of miR-301a-3p was detected in tumor tissues compared to those in corresponding non-tumor tissues (Table S1, detailed ISH evaluation listed in the Materials and Methods). Table 1 listed the characteristics of 90 PDAC patients and their relationships with the miR-301a-3p expression level. High expression of miR-301a-3p was significantly associated with lymph node metastasis(pN stage), advanced pathological stage(pTNM stage), but not with other clinical parameters. Since miR-301a-3p expression level was positively correlated to pathological features, we postulated that deregulation of miR-301a-3p may pose a promoting impact on PDAC progression and function as a predictor for poor prognosis. Kaplan-Meier analysis disclosed that parameters of significant prognostic influence on patients’ survival included pN stage, pTNM stage, in addition to miR-301a-3p expression (Table 2). Figure 1 illustrated the overall cumulative survival curves of low and high expression groups of miR-301a-3p using ISH score as the cut-off points (Figure 1a, detailed evaluation listed in the Materials and Methods). The three-year survival rate of the low expression group was significantly better than that of the high one(45.5% vs 19.5%, *p* = 0.007, Figure 1b).

**Table 1:** Clinicopathological correlations of miR-301a-3p expressions in 90 pancreatic cancer patients after pancreaticoduodenectomy

Parameters	High expression(*n* = 46)	Low expression(*n* = 44)	*P* value
Gender			0.953
Male	29	28	
Female	17	16	
Age			0.387
< 60	23	18	
≥ 60	23	26	
pT stage^a^			0.490
T1	4	1	
T2	34	36	
T3	8	7	
pN stage			0.031
N0	21	30	
N1	25	14	
pTNM stage^a^			0.038
I	16	24	
IIA	3	6	
IIB	27	14	
Perineural invasion			0.696
No	28	25	
Yes	18	19	
Lymphatic invasion			0.136
No	42	44	
Yes	4	0	
^§^Tumor volume(cm^3^)	24.9(1,324)	36(1.5, 280)	0.143

§ Data presented as median (range)

a There is no T4 stage tumor or M1 patients in our tissue microarray.

**Table 2:** Univariate and multivariate analysis of different prognostic parameters in 90 pancreatic cancer patients after pancreaticoduodenectomy

Parameters	Category	No.	Univariate		multivariate	
			3-year OS (%)	P	RR(95% CI)	*P* value
Age				0.806		
	<60	41	29.3			
	≥60	49	34.7			
Sex				0.082		
	Male	57	26.3			
	Female	33	42.4			
pT stage^a^				0.887		
	T1	5	20.0			
	T2	70	31.4			
	T3	15	40.0			
pN stage				0.003		0.016
	N0	41.2			1	
	N1	20.5			1.855(1.123 to 3.965)	
pTNM stage^a^				0.004		
	I	40	42.5			
	IIA	9	35.7			
	IIB	41	19.5			
Perineural invasion				0.525		
	No	53	37.7			
	Yes	37	24.3			
Lymphatic invasion				0.234		
	No	86	33.7			
	Yes	4	0.0			
Tumor volume(cm^3^)						
	≤ 50	57	31.6	0.617		
	<50	33	39.4			
miR-301a-3p expression				0.007		0.037
	Low	44	45.5		1	
	High	46	19.6		1.733(1.033 to 2.907)	

a There is no T4 stage tumor or M1 patients in our tissue microarray.

Furthermore, multivariate analysis through the Cox proportional hazard model was conducted to determine the independent prognostic value of miR-301a-3p expression level. The risk factors examined included miR-301a-3p expression level, pN stage and pTNM stage which could significantly reduce the survival outcome of PDAC patients (Table 2). Finally, high level of miR-301a-3p expression(RR 1.733; 95% CI 1.033 to 2.907; *p* = 0.037) as well as pN stage(RR 1.855; 95% CI 1.123 to 3.965; *p* = 0.016) in tumors was associated with a poor prognosis independent of other clinical covariates (Table 2).

### The expression of miR-301a-3p was up-regulated in PDAC samples and cell lines

**Figure 2:**
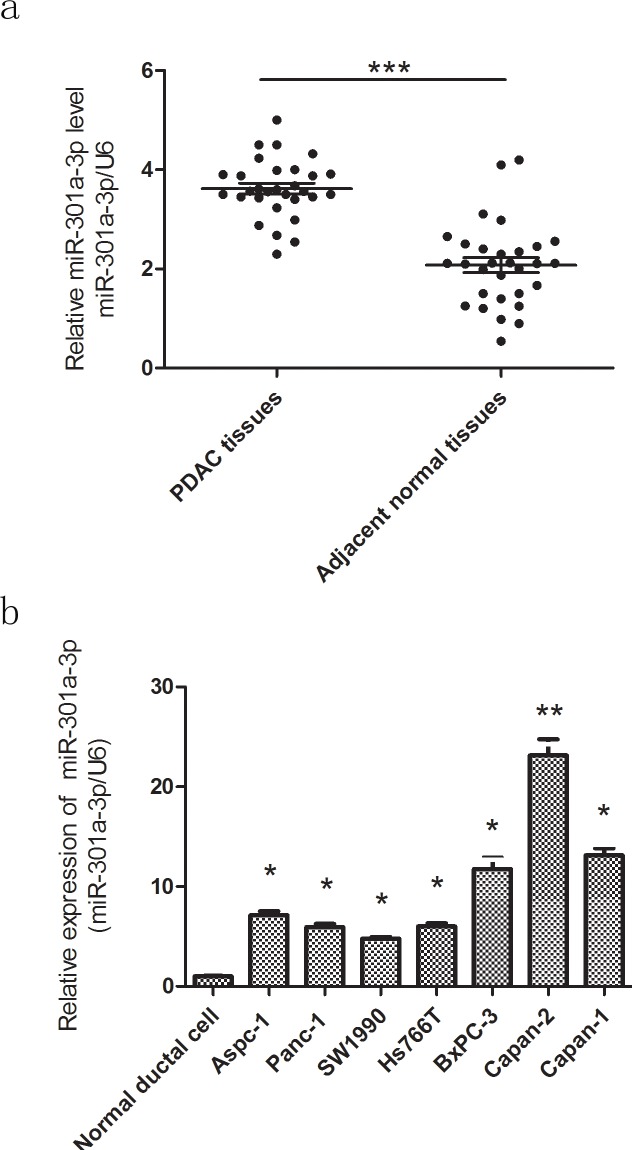
MiR-301a-3p is up-regulated in PDAC tissues and cell lines **a.** The expression levels of miR-301a-3p were examined by qRT-PCR in PDAC tissues and matched adjacent normal tissues of 30 patients. Transcripts levels were normalized to U6 expression. **b.** MiR-301a-3p expression in seven PDAC cell lines versus normal ductal cell. Data are shown as mean ± SEM of three independent experiments. *P < 0.05, **P < 0.01, ***P < 0.001.

In order to confirm miR-301a-3p expression in PDAC samples, qRT-PCR analysis was performed in PDAC tissues from 30 patients who underwent pancreaticoduodenectomy in Shanghai First People's Hospital. These results verified that the expression of miR-301a-3p was higher in PDAC tissues compared to matched adjacent normal tissues (Figure 2a). We also observed that miR-301a-3p was relatively higher in a series of tested human PDAC cell lines as compared with that found in normal human pancreatic duct epithelial cells(Figure 2b).

### MiR-301a-3p promoted colonogenicity, invasion and migration in PDAC cell lines but had no effects on proliferation *in vitro*

**Figure 3:**
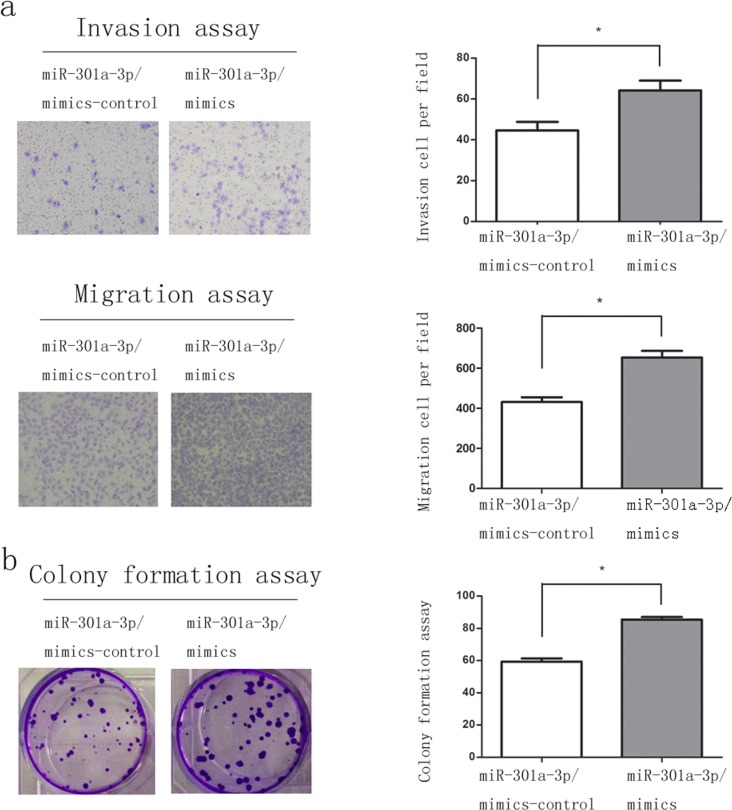
Overexpression of miR-301a-3p enhance clonogenicity, migration and invasion of sw1990 cells **a.** Representative fields of miR-301a-3p/mimics-control and miR-301a-3p/mimics cells in the invasion and migration assays and average number of invasion and migration cells per field was evaluated. **b.** Representative micrographs of soft agar colony formation assay of the indicated cells. The colonies were counted and identified. Data are shown as mean ± SEM of three independent experiments. *P < 0.05, **P < 0.01.

**Figure 4:**
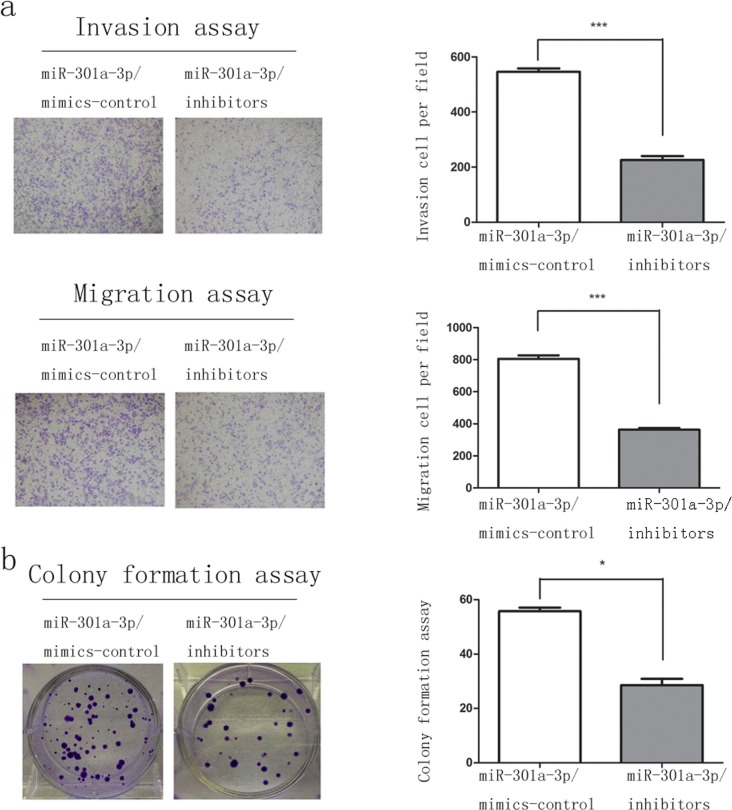
MiR-301a-3p knockdown inhibit clonogenicity, migration and invasion of Capan-2 cells **a.** Representative fields of miR-301a-3p/inhibitors-control and miR-301a-3p/inhibitors cells in the invasion and migration assays and average number of invasion and migration cells per field was evaluated. **b.** Representative micrographs of soft agar colony formation assay of the indicated cells. The colonies were counted and identified. Data are shown as mean ± SEM of three independent experiments. *P < 0.05, ***P < 0.001.

We investigated the role of miR-301a-3p in the proliferation, clonogenicity, invasion and migration of PDAC cell lines, which were important aspects for tumorigenesis and metastasis. Considering subsequent overexpression and knockdown of miR-301a-3p, sw1990 and Capan-2 cells were selected for up-regulation and down-regulation investigation respectively, according to miR-301a-3p expression profiles in PDAC cell lines (Figure 2b). After transfecting sw1990 cells with miR-301a-3p mimics (sw1990-miR-301a-3p/mimics) and Capan-2 cells with miR-301a-3p inhibitors (Capan-2-miR-301a-3p/inhibitors) respectively, the matrigel invasion and migration assays confirmed that the overexpression of miR-301a-3p increased the invasiveness and migration of sw1990 cells compared with the control cells (Figure 3a). On the contrary, the inhibition of miR-301a-3p in Capan-2 cells reduced their motility (Figure 4a). In colony formation assays, the colony number of sw1990-miR-301a-3p/mimics was higher than their control group (Figure 3b); While Capan-2-miR-301a-3p/inhibitors showed lower colony forming ability compared with Capan-2- miR-301a-3p/inhibitors-control cells (Figure 4b). Then we performed CCK-8 assays to investigate the effects of miR-301a-3p on cells proliferation. As a result, there was no significant difference observed (Figure S1). These results indicated that miR-301a-3p enhanced PDAC cells motility and colonogenicity *in vitro*.

### *SMAD4* is a target gene of miR-301a-3p

**Figure 5:**
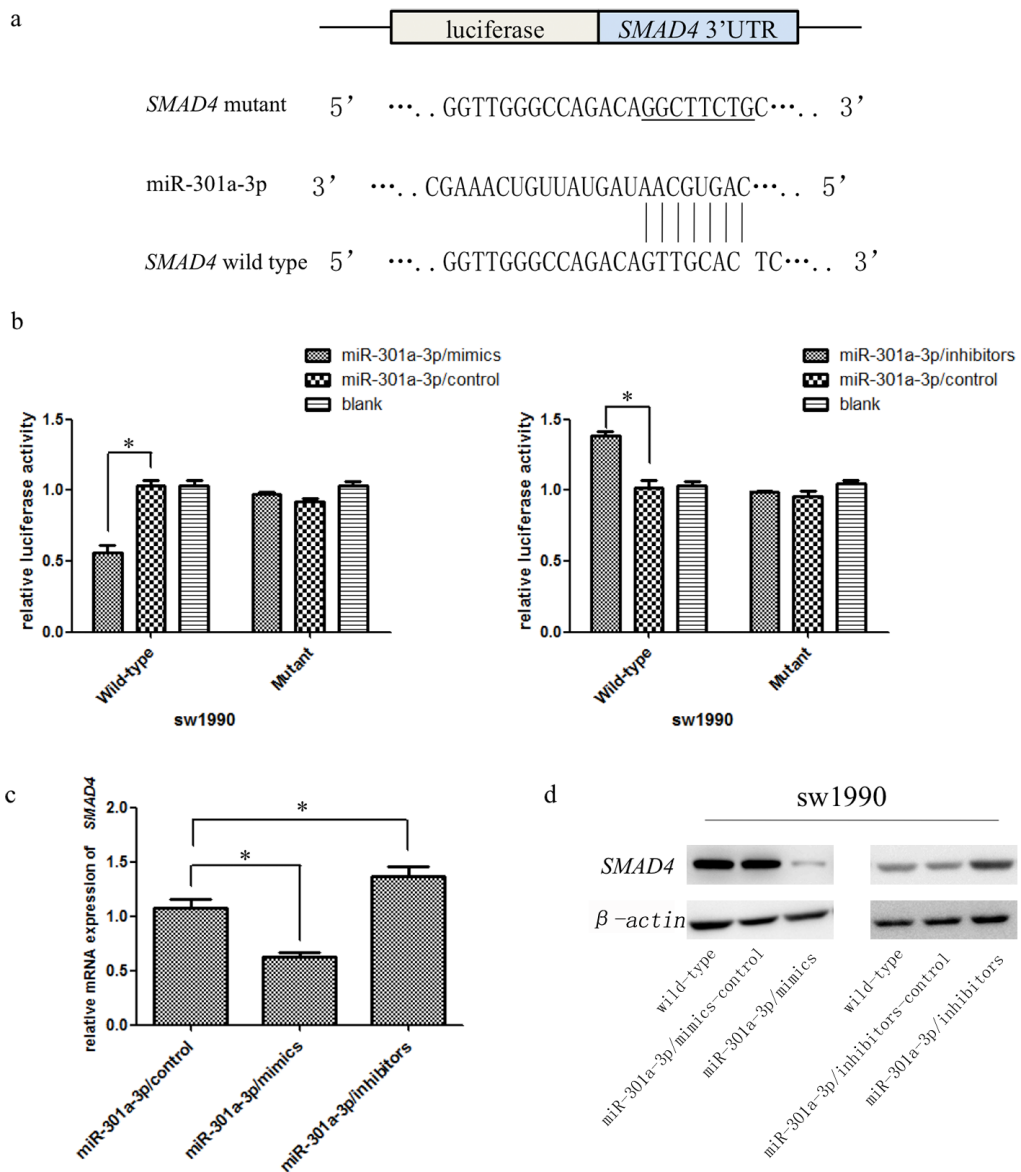
MiR-301a-3p decreased *SMAD4* expression by directly binding its 3′UTR **a.** The putative miR-301a-3p seed region at the 3′UTR of *SMAD4* mRNA was computationally predicted. A 3′UTR fragment of *SMAD4* mRNA containing wild type or mutant (underlined) of the miR-301a-3p binding sequence was cloned into downstream of the luciferase gene. **b.** Luciferase reporter assays in sw1990 cells with co-transfection of wild type or mutant *SMAD4* 3′UTR as well as either miR-301a-3p/mimics, miR-301a-3p/inhibitors or miR-301a-3p/control was performed. Luciferase activity was normalized by the ratio of firefly and Renilla luciferase signals and determined 48h after transfection. **c.** *SMAD4* mRNA was detected by qRT-PCT in sw1990 cells transfected with miR-301a-3p/control, miR-301a-3p/mimics or miR-301a-3p/inhibitors. **d.** *SMAD4* protein in sw1990 cells was examined by Western blot at 48h post-transfection with miR-301a-3p/control, miR-301a-3p/mimics or miR-301a-3p/control, miR-301a-3p/inhibitors and compared to wild-type cells. Data are shown as mean ± SEM of three independent experiments *P < 0.05.

To deeply explore the mechanisms of miR-301a-3p in PDAC progression, we searched for its candidate target genes by bioinformatical analysis. StarBase, TargetScan and miRBase were utilized for searching. Among these predicted targets, tumor suppressor gene *SMAD4* was identified as one of the potential target genes of miR-301a-3p in PDAC cells (Figure 5a). To validate whether the 3′UTR of *SMAD4* mRNA was a functional target of miR-301a-3p, luciferase reporter gene assays were performed. The 3′UTR sequence of *SMAD4* (wt 3′UTR) or the mutant sequence (mut 3′UTR) was cloned into a luciferase reporter vector, along with the pRL-TK plasmid containing the Renilla luciferase gene as an internal control. Luc-*SMAD4*-wt co-transfected with MiR-301a-3p/mimics in sw1990 cells showed a significant decrease of luciferase activity compared with control group. However, Luc-*SMAD4*-mut plasmid co-transfected with miR-301a-3p/mimics showed no significant difference in reporter activity compared with co-transfected with miR-301a-3p/mimics-control cells. Likewise, miR-301a-3p/inhibitors increased the luciferase activity of Luc-*SMAD4*-wt but had no effect on Luc-*SMAD4*-mut plasmid (Figure 5b). Moreover, overexpression of miR-301a-3p significantly inhibited *SMAD4* mRNA and protein levels in sw1990 cells, while inhibition of miR-301a-3p revealed opposite effects (Figure 5c, 5d).

### *SMAD4* acts as a tumor suppressor

**Figure 6:**
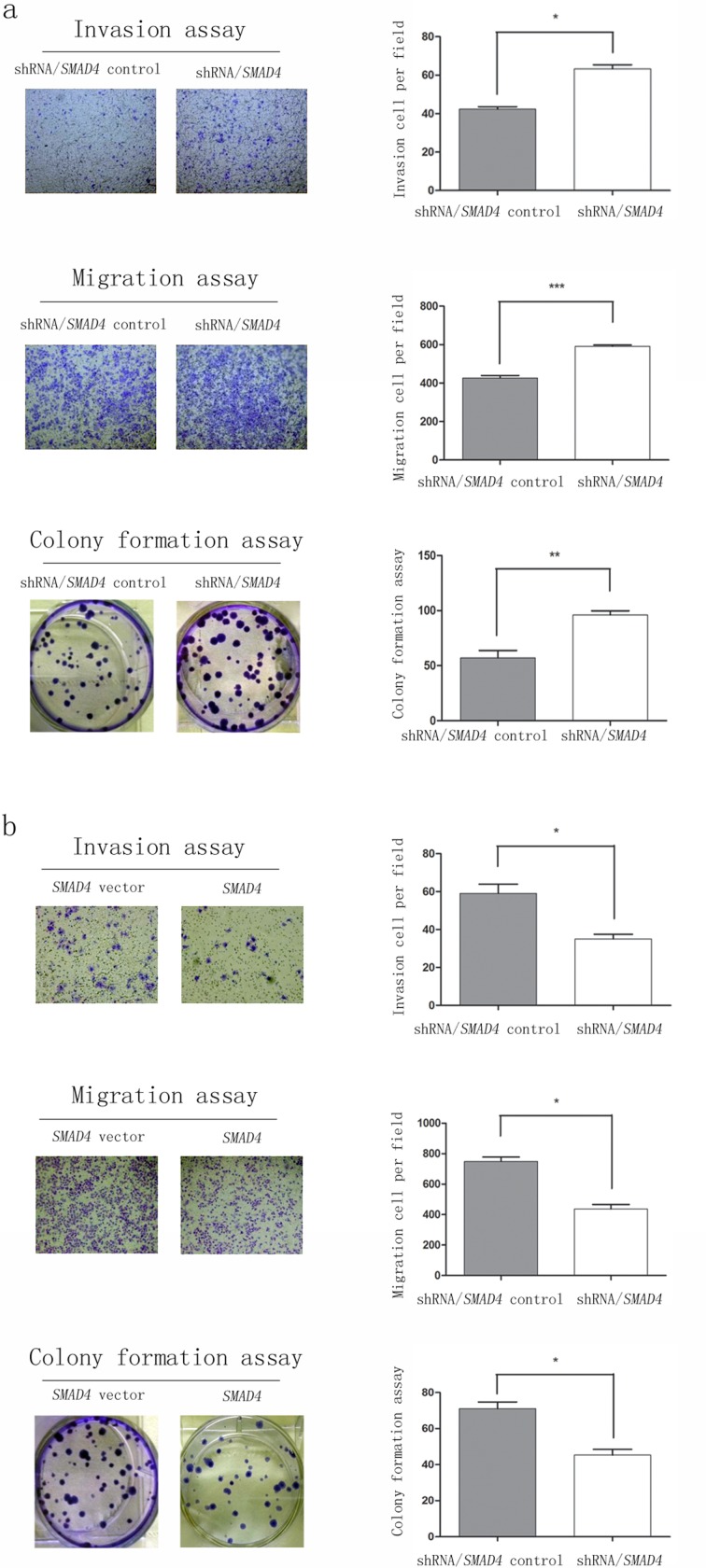
*SMAD4* acts as a tumor suppressor after establishing sw1990 cell lines up-regulation or down-regulation *SMAD4* gene Colony, migration and invasion assays of sw1990 cells were performed after **a.** *SMAD4* knockdown (control or shRNA/*SMAD4*) and **b.** overexpression (vector or *SMAD4*). All assays have been performed three times independently and data are presented as mean ± SEM. *P < 0.05, **P < 0.01, ***P < 0.001.

*SMAD4* gene inactivation had been reported to be associated with poorer prognosis in patients with surgically resected PDAC [18, 19]. A few studies had shown that *SMAD4* was more inclined to behave as a tumor suppressor [20, 21]. However, the effects of *SMAD4* on PDAC cells are not yet well established. In order to further investigate its specific functions, we induced *SMAD4* overexpression and knockdown in sw1990 cell lines. *SMAD4* expression was evidently up-regulated or down-regulated as confirmed with Western blot (Figure S2). *SMAD4*-depleted cells exhibited significant increased colony formation, invasion and migration abilities, relative to those transfected with control vector (Figure 6a). Conversely, overexpression of *SMAD4* led to significantly decreased colony, invasion and migration abilities (Figure 6b). Those data collectively indicated a tumor suppressing function of *SMAD4* in PDAC cells.

### Restoration of *SMAD4* attenuated miR-301a-3p induced PDAC cell colony, invasion and migration

**Figure 7:**
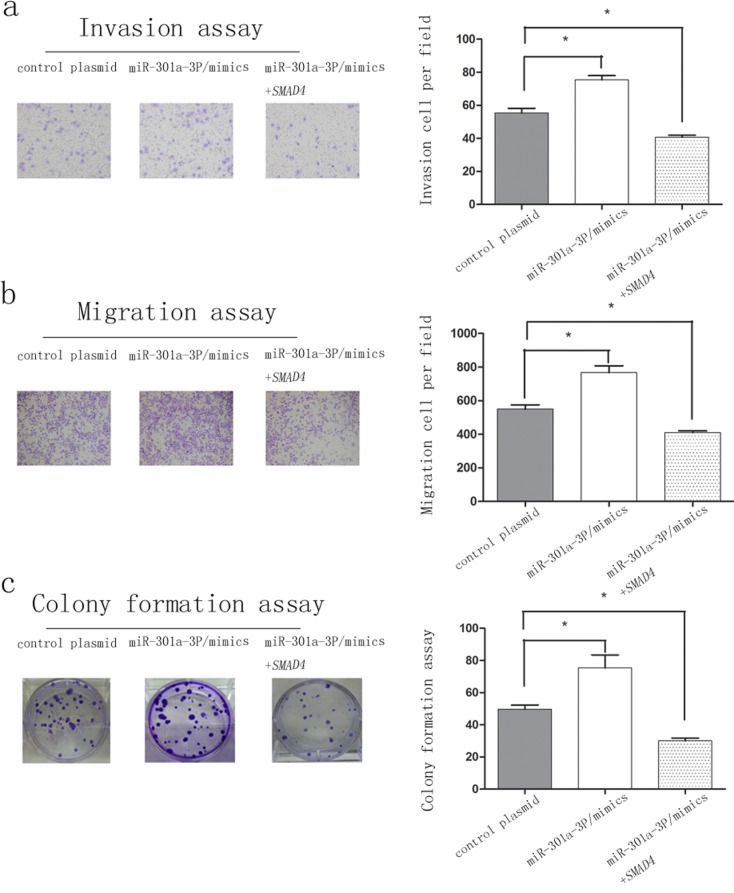
*SMAD4* attenuated miR-301a-3p mediate colony formation, migration and invasion of sw1990 cells The invasiveness **a.**, migration **b.** and colony formation **c.** abilities are detected in cells transfected with control plasmid, miR-301a-3p/mimics and miR-301a-3p/mimic+*SMAD4*. All assays have been performed three times independently and data are presented as mean ± SEM. *P < 0.05, **P < 0.01.

To further determine whether miR-301a-3p promoted colony formation, migration and invasion is ascribable to *SMAD4* gene. Sw1990 cells were co-transfected with miR-301a-3p mimics+*SMAD4*, miR-301a-3p mimics or control plasmid. As suggested in Figure 7, overexpression of miR-301a-3p leading to significant enhancements in cell invasion (Figure 7a), migration (Figure 7b) and colony formation (Figure 7c) was significantly suppressed by restoration of *SMAD4* expression. Hence, concurrent repression of *SMAD4* appeared to play a pivotal role in miR-301a-3p induced cell colonogenicity, migration and invasion.

### MiR-301a-3p promotes tumorigenicity via repression of SMAD4 *in vivo*

**Figure 8:**
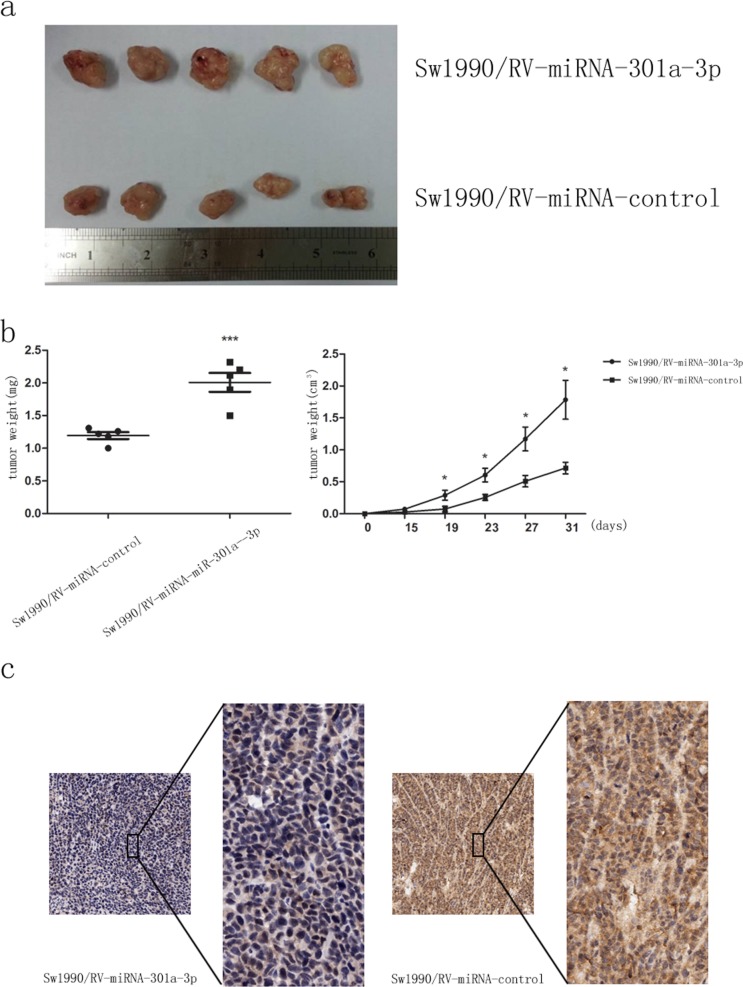
MiR-301a-3p promotes tumorigenicity *in vivo* **a.** Photographs of tumors derived from sw1990/RV-miR-301a-3p and sw1990/RV-miR-control cells in nude mice. **b.** The graphs are representatives of tumor growth 31 days after inoculation. **c.** Representative photographs of immunohistochemical analysis of *SMAD4* antigen in tumors of nude mice. Clearly, miR-301a-3p level was negatively correlated with that of *SMAD4*. Tumor volume and weight were calculated and all data are shown as mean ± SEM. *P < 0.05, ***P < 0.001.

Given that miR-301a-3p enhanced colonogenicity, migration and invasion *in vitro*, we examined whether miR-301a-3p could increase tumorigenicity *in vivo*. Retrovirus-mediated sw1990/ miR-301a-3p and sw1990/ miR-control stable cell lines were cultured as described in the Methods. Expression of miR-301a-3p in stably transfected with miR-301a-3p sw1990 was about 2.5 times higher than that of miR-control group (Figure S3). Sw1990/RV-miR-301a-3p and sw1990/RV-miR-control cells were then injected subcutaneously into four-week-old female nude mice respectively and tumor volume and weight were monitored (Figure 8a). Tumors grew more rapidly in the sw1990/ RV-miR-301a-3p group than those in the sw1990/ RV-miR-control group (Figure 8b). The average tumor volume and weight in mice inoculated with sw1990/ RV-miR-301a-3p at 31 days was significantly larger than mice inoculated with sw1990/ RV-miR-control (Figure 8b). In order to assess whether tumor growth promotion in sw1990/ RV-miR-301a-3p cells was partly due to the suppression of *SMAD4*, immunohistochemical analysis of xenograft tumor tissues were performed. As shown with *SMAD4* antigen staining (Figure 8c), the tumors from the sw1990/ RV-miR-301a-3p group expressed less *SMAD4* than those from sw1990/ RV-miR-control group, that supported our hypothesis that the increased tumor growth in mice injected sw1990/ RV-miR-301a-3p cells might be partially because of inhibition of *SMAD4* expression caused by the up-regulated miR-301a-3p. These data together further showed an inverse correlation of *SMAD4* protein with miR-301a-3p expression in PDAC even *in vivo* experiments.

### Downregulation of *SMAD4* inversely correlate with miR-301a-3p expression in PDAC tissues

**Figure 9:**
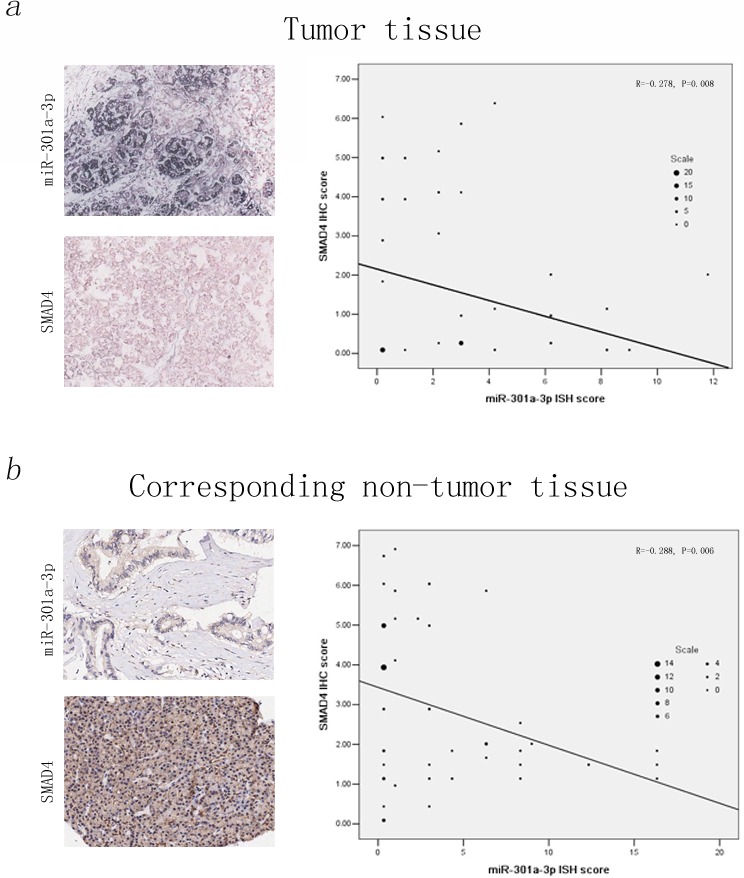
Downregulation of *SMAD4* is inversely correlated with miR-301a-3p expression in the tissue microarray of 90 PDAC samples The relationship between miR-301a-3p expression and *SMAD4* protein levels were determined using ISH and IHC score, respectively (Detailed ISH and IHC evaluation had been listed in the Materials and Methods). Clearly, miR-301a-3p expression level was negatively correlated with that of *SMAD*. A statistically significant inverse correlation was observed between miR-301a-3p and *SMAD4* in tumor tissues (a, Pearson's correlation analysis, R =− 0.278, *P* = 0.008) and corresponding non-tumor tissue (b, Pearson's correlation analysis, R =− 0.288, *P* = 0.006).

Next, we investigated the *SMAD4* protein levels in 90 PDAC samples by IHC method. A significant low level IHC scores of *SMAD4* was examined in tumor tissues compared to those in corresponding non-tumor tissues (Table S2, detailed IHC evaluation listed in the Materials and Methods). Furthermore, when we compared the expression levels of *SMAD4* and miR-301a-3p using Pearson's correlation analysis, a significantly inverse correlation between miR-301a-3p ISH scores and *SMAD4* IHC scores were observed in those 90 PDAC paired tissues, indicating that the overexpression of miR-301a-3p and *SMAD4* silencing of might jointly contribute to PDAC development (Figure 9).

## DISCUSSION

In just a few short years, accumulating evidence has emerged to support the important roles of miRNAs as key molecular components of the cell in both normal and pathologic states [22]. In particular, cancer has been a major field of miRNA research over the past decades and several studies demonstrated that through controlling expression of their target mRNAs, miRNAs could facilitate tumor growth, invasion, angiogenesis and even immune evasion [23, 24]. Hence, in this study we focused on the biological roles and detailed mechanisms of miR-301a-3p in PDAC tumorigenesis.

According to MiRBase (http://www.mirbase.org/index.shtml), has-miR-301a-3p is located on human chromosome 17q22, overlapping the SKA2 gene. It had been reported previously that miR-301a-3p acted as an oncogene in many tumors, e.g. gastric cancer, non-small cell lung cancer and breast cancer. These studies showed that miR-301a-3p was involved in different molecular mechanisms, including the PTEN/Akt signaling pathway, the Wnt pathway and the ERK/CREB pathway [13, 15; 25]. Other researchers have even found that miR-301a-3p may also contributed to inflammation development [26], implying that it has a central role in regulating several signaling pathways. In pancreatic cancer a microRNA expression profiling study had been mentioned that miR-301a-3p was overexpression [27] and Lu et al. found that there was a positive feedback loop in tissues between miR-301a-3p and NF-κB in which miR-301a-3p repressed NF-κB-repressing factor to elevate NF-κB activity that in turn promoted miR-301a-3p transcription [28]. However, the biological behaviors and mechanisms that were responsible for the carcinogenesis remain undefined. Our current results showed that miR-301a-3p expression was up-regulated in PDAC tissues and cell lines compared with matched adjacent tissues and normal cells, thus raising the possibility that the expression of miR-301a-3p could be a useful diagnostic biomarker for PDAC. Indeed, in our microarray analysis, ISH results revealed that miR-301a-3p was a significant predictor for advanced clinic stage, lymph node metastasis and even poorer overall survival in PDAC patients. This is similar to the role of miR-301a-3p in gastric cancer [29] and breast cancer [13]. Subsequently, *in vitro* experiments miR-301a-3p overexpression promoted PDAC cells colony, invasion and migration abilities, whereas its depletion led to opposite effects. Consistent with the results of several other kinds of cancer studies for miR-301a-3p, this observed phenotype further suggests that miR-301a-3p is a candidate oncogene of PDAC. While Lu et al. [28] demonstrated that miR-301a-3p inhibition reduced xenograft tumour growth, in our mouse subcutaneous xenograft model up-regulating miR-301a-3p promoted tumor formation and growth which both supported miR-301a-3p tumorigenesis role.

MiRNAs bind to perfect or imperfect complementary “seed” sequences in target mRNAs, leading to cleavage of target mRNAs or inhibition of their translation [30]. One miRNA potentially targets different mRNAs. The miR-301a-3p had been reported to target MEOX2 in lung cancer [25], FOXF3, BBC3, PTEN and COL2A1 in breast cancer [13] and RUNX3 in gastric cancer [14]. While in our study, *SMAD4* was identified as a potential target gene. Firstly, overexpression of miR-301a-3p decreased the activity of a luciferase reporter gene including the 3′UTR of *SMAD4*, while mutation of the “seed region” sites in the 3′UTR of *SMAD4* abolished the regulatory effect of miR-301a-3p. The opposite effect was observed when we co-transfected miR-301a-3p/inhibitors. Secondly, overexpression of miR-301a-3p down-regulated *SMAD4* at the mRNA and protein levels in pancreatic cancer cell lines, and vice verse. Thirdly, there is a functional overlap between miR-301a-3p and *SMAD4* in colony, invasion and migration assays. Last but not the least, in tumor xenograft samples and clinical samples IHC analysis, enforced miR-301a-3p overexpression triggered a repressive effect on endogenous *SMAD4* protein expression. All these results indicated that *SMAD4* was supposed to be a direct target gene of miR-301a-3p in PDAC, which were consistent with what Lin et al. found in human colorectal cancer [31]

*SMAD4* has been documented in a large number of cancers [32], including PDAC [33], in which this gene was more likely considered as a kind of tumor suppressor in tumor growth [34], metastasis and relapse [35, 36]. This tumor suppressor gene, encoding a transcription factor, is a central mediator of the transforming growth factor-β(TGF-β) signal pathway. Binding of the TGF-β to its receptor leads to phosphorylation of *SMAD2*/*SMAD3* to form a complex with *SMAD4* for stimulation or repression of target genes that cause cell cycle arrest and apoptosis of epithelial cells [37]. Moreover, in patients, it is especially inactivated in over half of invasive PDACs [38], which means this key gene is relatively specific for PDAC. In benign epithelia and early stage of tumor, TGF-β is a potent inducer of growth arrest. However, in advanced stage, Rather than inhibiting carcinogenesis, TGF-β promotes tumor progression. This functional switch is known as the TGF-β paradox [39]. In other words, advanced PDAC cells have lost their tumor-suppressive effectors but possessed tumor-promoting effectors induced by increased TGF-β. Levy et al [20] used a tetracycline-inducible small interfering RNA approach to inhibit *SMAD4* function in PDAC cell line Colo-357 and demonstrated that loss of *SMAD4* indeed promoted TGF-β mediated tumorigenesis through abolishing tumor-suppressive functions of TGF-β/*SMAD4*. Therefore, our findings about miR-301a-3p's effect on *SMAD4* could be a possible explanation for *SMAD4* inactivation in PDAC progression. Whereas, based on most of *SMAD4* inactivated in later stage of PDACs, further works should focus on whether miR-301a-3p expression is associated with *SMAD4* expression in terms of different tumor stages.

Although in our results we demonstrated that overexpression of miR-301a-3p repressed *SMAD4* expression through direct binding to its 3′UTR, there were still other miRNA consensus in the *SMAD4* 3′UTR (e.g., sites for miR-34a, miR-146a, and miR-199a, which have been identified as negative regulators of *SMAD4* in gastric cancer and glioblastoma) [40, 41, 42]. Therefore, we could not rule out the possibility that these miRNAs also participate in the down-regulation of *SMAD4* expression in the development of PDAC. Also, we cannot exclude the possibility that other potential targets of miR-301a-3p may govern additional cancer pathways as well as promoting the development of PDAC, as a single miRNA is known to target multiple mRNAs. These hypotheses indicate the need for further studies to reveal the entire “targetome” of the miR-301a-3p in pancreatic carcinogenesis and progression.

Another limitation is the difference between the results of miR-301a-3p or *SMAD4* manipulated animals or cell lines and that of clinicopathological variables such as perineural invasion, lymphatic invasion or tumor volume (Tables 1 and 2). However, this discrepancy might result from that in patients’ clinical data, those clinicopathological characteristics were determined by multiple factors, such as age, hormones level, operation, pathological examination and even inevitable biases. Conversely, pancreatic cancer cell lines were comparatively stable to manipulate in medium. Secondly, it is not acceptable to implement duodenopancreatectomy for an advanced stage tumor if the operation would increase the patient's risk or decrease survival. That was why in our 90 clinical specimens there were no III and IV stage patients who might show higher rate of perineural invasion, lymphatic invasion and tumor volume. However, currently our surgical team had been collecting more PDAC samples and further analysis about this issue would be done in our future work.

Finally, the present study examined the role and underlying mechanism of miR-301a-3p in pancreatic cancer. The question that whether miR-301a-5p has the same role and mechanism as miR-301a-3p is also worthy of a deep investigation in pancreatic cancer. However, our research team had not collected enough data about miR-301a-5p so that we could hardly show result about it.

In summary, our results show that miR-301a-3p, important onco-miRNA, are up-regulated in both PDAC tissues and cell lines. Ectopic expression of miR-301a-3p stimulates PDAC cell lines colony, invasion and migration through direct targeting of *SMAD4*. We also showed that miR-301a-3p could be a significant biomarker for lymph node metastasis, advanced pathological stage and poor prognostic predictor in PDAC. Therefore, miR-301a-3p inhibitory oligonucleotides or overexpression of *SMAD4* may have therapeutic potential to suppress PDAC progression. Further researches on miR-301a-3p are warranted, in view of their potential as diagnostic and therapeutic agents.

## MATERIALS AND METHODS

### Ethical statement

Written informed consent was obtained from all participants and study protocol was approved by the ethics committee of Shanghai First People's Hospital affiliated of Shanghai Jiaotong University and conducted in full accordance with ethical principles, including the World Medical Association Declaration of Helsinki and local legislation. All mouse experiments were manipulated and housed according to protocols approved by Shanghai Medical Experimental Animal Care Commission.

### Human tissue specimens and cell lines

Thirty pairs of PDAC specimens and their matched non-tumor adjacent tissues were obtained from patients undergoing surgery for PDAC at Shanghai First People's Hospital affiliated of Shanghai Jiaotong University. All tissues were immediately snap-frozen in liquid nitrogen and stored at − 80 °C until DNA and RNA extraction. The study was approved by the Ethics Committee of Shanghai First People's Hospital. The human pancreatic cancer cells, including Aspc-1, Panc-1, sw1990, Hs766T, BxPC-3, Capan-2 and Capan-1 were obtained from the Americacn Type culture Collection (ATCC, manassas, VA) and the normal human pancreatic duct epithelial cells were isolated from normal pancreatic tissues as described [16]. Panc-1, sw1990, Hs766T, Capan-2 and normal human pancreatic duct epithelial cells cell lines were maintained in DMEM with 10% FBS (GIBCO, Carlsbad, CA). Aspc-1, BxPC-3 cell lines were maintained in 1640 with 10% FBS. Capan-1 cell line were maintained in IMDM with 10% FBS. All cells were fostered in a humidified atmosphere of 5% CO_2_ and 95% air.

### RNA isolation and quantitative real-time PCR (qRT-PCR)

Total RNA extraction from tissues samples and cell lines used TRIzol reagent (Invitrogen, Carlsbad, CA) according to the manufacturer's instructions. Complementary DNA was synthesized with the Prime-Script RT reagent kit (Promega, Madison, WI). Real-time PCR was performed using SYBR Green PCR Master Mix (Applied Biosystems, Foster City, CA) on an ABI 7900HT fast real-time PCR system (Applied Biosystems). Expression data were uniformly normalized to the internal control U6 and the relative expression levels were evaluated using the ΔΔCt method. The primers for *SMAD4* were 5′-GCTGCTGGAATTGGTGTTGATG-3′ (forward) and 5′-AGGTGTTTCTTTGATGCTCTGTCT-3′ (reverse) and for GAPDH were 5′-GGACCTGACCTGCCGTCTAG-3′ (forward) and 5′-GTAGCCCAGGATGCCCT TGA-3′ (reverse) according to the human *SMAD4* and GAPDH cDNA sequences in GenBank. The GAPDH mRNA level was used for normalization. PCRs of each sample were conducted in triplicate.

### Transient transfection

The hsa-miR-301a-3p/mimic, mimic negative control, hsa-miR-301a-3p/inhibitor and inhibitor negative control oligonucleotides were purchased from GenePharma. Cells in logarithmic growth phase were trypsinized, counted, and seeded in 6-well plates to ensure 50% cell confluence on the next day for transfection. Transfection of cells with oligonucleotides was performed using Lipofectamine™ 2000 Reagent in line with the manufacturer’ instructions (Invitrogen) at a final concentration of 100 nM.

### Luciferase assays

About 1 × 10^5^ sw1990 cells per well were seeded in 24-well plates for 24h before transfection. About 100 ng of wild-type or mutant *SMAD4* 3′-UTR psiCHECK-2 plasmid (Promega) was transiently cotransfected with 60 pmol miR-301a-3p/mimic or control and miR-301a-3p/inhibitor or control into sw1990 cells using 1.44μl Lipofectamine reagent (Invitrogen). Cell lysates were harvested 48 h after transfection and then firefly and Renilla luciferase activities were measured by the Dual-Luciferase Reporter Assay System (Promega) on a Berthold AutoLumat LB9507 rack luminometer. Renilla luciferase activities were normalized to firefly luciferase activities to control for transfection efficiency.

### Cell migration and invasion assays

Cell migration and invasion ability were examined by Corning transwell insert chambers (8 mm pore size; Corning) and BD BioCoat Matrigel Invasion Chamber (BD Biosciences, Bedford, MA), respectively. The chemoattractant was 10% FBS. Cells were harvested and resuspended in serum-free medium after transfection. About 2 × 10^4^ (migration assay) or 6 × 10^4^ (invasion assay) prepared cells were added into the chamber and incubated for 24 h at 37°C. Cells were suspended in serum-free medium and placed in the top chambers and complete medium containing 10% FBS was added into the bottom chambers. After 24h incubation, the noninvasive cells were gently removed from the top wells with cotton-tipped swab and cells that had migrated or invaded through the membrane were fixed with 20% methanol and stained with 0.1% crystal violet (Invitrogen), then imaged and counted.

### Colony formation assay

Five hundred indicated sw1990 cells were placed in complete growth media and allowed to grow until visible colonies formed in a fresh six-well plate (2 weeks at most). Cell colonies were fixed with cold methanol, stained with 0.1% crystal violet for 30 min, washed, air dried, photographed and counted.

### Cell proliferation assay

Cell proliferation assay was performed with Cell Counting Kit-8 (Dojindo, Kumamoto, Japan) according to the manual of the manufacturer. Briefly, indicated PDAC cells were seeded in 96-well plates (1 × 10^4^ cells/well) 24 h post-transfection and cultured in the growth medium. Cells were examined at 0h, 24h, 48h, 72h. CCK-8 (10 μl) was added to each well at different time points. After an incubation of 2 h at 37°C, absorbance was measured at 450 nm.

### Western blot analysis

Whole cell protein lysates were electrophoresed on 10% sodium dodecyl sulfate-polyacrylamide gels and transferred onto polyvinylidene difluoride membranes (Millipore). The membranes were incubated with primary antibodies overnight at 4°C and then with the appropriate horseradish peroxidase-conjugated secondary antibody. The following antibodies were used: β-actin (CP01, Calbiochem, San Diego, CA), *SMAD4* antibody (ab40759, abcam, Cambridge, UK).

### Retroviral transduction for stable cell lines

Packaging of pseudotyped recombinant lentivirus was performed by transfection of 293T cells. Briefly, miR-301a-3p-overexpressed vector pLV-mCherry(2A)puro-miR301a-3p or control vector pLV-mCherry(2A)puro was co-transfected with package vectors psPAX2 and pMD2.G into 293T cells using Lipofectamine 2000 (Invitrogen, Paisley, Scotland, UK). Lentivirus in the culture media was harvested at 72 h and filtered through a 0.45 μm low protein binding polysulfonic filter (Millipore, Bedford, MA). The virus was frozen and kept in −70°C refrigerator. For virus infection, sw1990 cells were plated into 6-cm dishes in advance and cultured for nearly 18 hours with about 50% confluence before transduction. The lentivirus suspension in the presence of 8 μg/ml polybrene (Chemicon, Temecula, CA) was added to the cells.

### Tumor xenograft model and tumorigenicity assay

Sw1990 cells 2*10^6^ cells/mouse stably transfected with retrovirus-miR-301a-3p or retrovirus-control vector were subcutaneously injected into 4-week-old female nude mice. Mice were checked weekly, and tumor nodules were measured with a caliper every 4 days. Tumor volume was evaluated using the following formula: volume = (width + length)/2 × width × length × 0.5236. Tumor growth curves were calculated. The two experimental groups were sacrificed after 5 weeks. All tumor grafts were excised, weighed, harvested, fixed, and embedded.

### Tissue microarray construction

Pancreatic cancer samples with informed consent were collected between 1995 and 2009 from 90 patients who underwent pancreatic surgery and were were stored at Biobank Center of National Engineering Center for Biochip at Shanghai. All patients including complete clinical data and adequate tissue in this study were identified. Ethical approval for the study was obtained from the ethical committee of biobank center related hospitals.

Original formalin-fixed, paraffin-embedded specimens were used to construct a PDAC tissue microarray (FFPE TMA). Hematoxylin and Eosin (H&E)-stained standard slides from each tumor specimen were reviewed by a single pathologist (MR), who was blinded to specimen protein expression status. Representative tumor regions and its paracancerous nonmalignant pancreatic specimens (NMPs) were selected from each tissue block and 2 tissue cores (0.6 mm in diameter) were taken from each region using an automated tissue arrayer (Beecher Instruments, Sun Prarie, WI). Cores were transferred to individual recipient blocks. In all cases, cores were taken normal adjacent pancreas were also used as internal controls. Five-micron sections were cut from each recipient block. Sections were stained with H&E to confirm the presence of tumor within each core.

### *In situ* hybridization(ISH) and Immunohistochemistry(IHC)

PDAC tissues for ISH were fixed in 4% paraformaldehyde, dehydrated in a graded series of ethanol baths and embedded in paraffin. Then tissue slides were deparaffinized and digested with proteinase K for 30 min. After that, the slides were prehybridized in a hubridization solution at 57°C for 2 hours. Tissues were hybridized overnight in the presence of 10ng 3′-5′ DIG-labeled miR-301a-3p LNA probes at 50°C. Slides were washed twice stringently and an immunological reaction was carried out by using the rabbit antibody against digoxingenin and alkaline phosphatase, according to the manufacturer's recommendation. Each side was assigned a score for intensity and staining positive pattern.

To visualize xenograft tumor, the mice were killed. The tumors were dissected and fixed in 4% paraformaldehyde before paraffin embedding. The tissue was then sliced as 4μm sections and stained in hematoxylin and eosin. After deparaffinization and rehydration, antigen retrieval was performed by boiling in 10 mmol/l of citrate buffer (pH 6.0) for 10 min. After inhibition of endogenous peroxidase activity for 30 min with methanol containing 0.3% H_2_O_2_, the sections were blocked with 2% bovine serum albumin in PBS for 30 min and incubated with rabbit anti-human *SMAD4* monoclonal antibody(ab40759, abcam, Cambridge, UK). Images were captured at×40 magnification.

The percentage of positive tumor cells was set as Fang et al's methods [17]: 1 (up to 25% of positive cells), 2 (25% to 50% of positive cells), 3 (50% to 75% of positive cells) and 4 (more than 75% of positive cells). Intensity scores ranged from 0-3: 0, no staining; 1, weak staining; 2, moderate staining, and 3, strong staining. Multiplication of the two scores resulted in a final score ranging from 0 to 12. Under these conditions, samples with score 0-3 and score 4-12 were defined as low and high expression.

### Statistical analysis

Data were presented as mean ± SEM(parametric data) or median ± range (non-parametric data). Data were compared by using the student *t* test for paired and Mann-Whitney U test for unpaired continuous variables and the chi-squared or Fisher's exact test for discrete variables. Survival curves were estimated using the Kaplan-Meier method, and the log-rank test was used to calculate differences between the curves. Independent variables with a *P* value <0.05 in the univariate analysis were entered into the multivariate cox regression model. The SPSS 13.0 statistical software package was used for the analysis (SPSS Inc., Chicago, IL, USA). A *P* value <0.05 was considered significant.

## SUPPLEMENTARY MATERIAL FIGURES AND TABLES


