# Supplementary material for: MicroRNA-301a-3p promotes pancreatic cancer progression via negative regulation of SMAD4
Source: Oncotarget. 2015 May 12;6(25):21046–63. doi: 10.18632/oncotarget.4124 (PMC4673249; doi:10.18632/oncotarget.4124)
Supplement: Supplementary file 1 [file oncotarget-06-21046-s001.pdf]

MicroRNA-301a-3p promotes pancreatic cancer progression via negative regulation of *SMAD4*

Supplementary Material

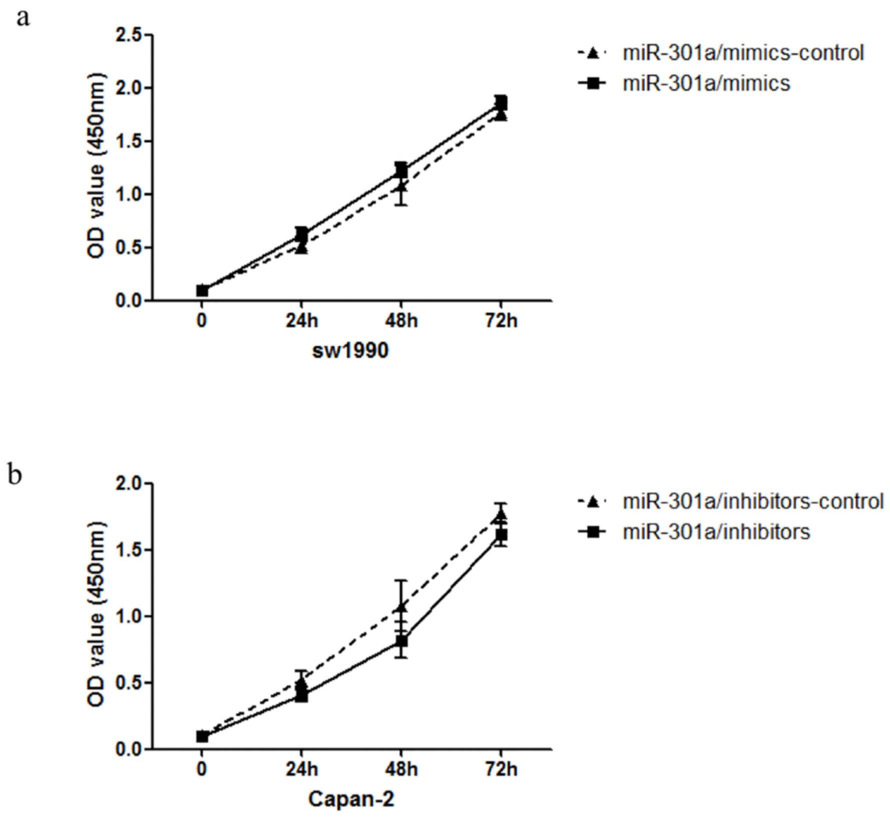

Figure S1: Role of miR-301a-3p in proliferation. Cell proliferation was measured by CCK-8 assay in sw1990 and Capan-2 cell lines transfected with miR-301a-3p mimics, inhibitors or negative control. The X-axis indicates the number of hours after transfection. Data represent the mean ± SEM of the three independent experiments performed in triplicate.

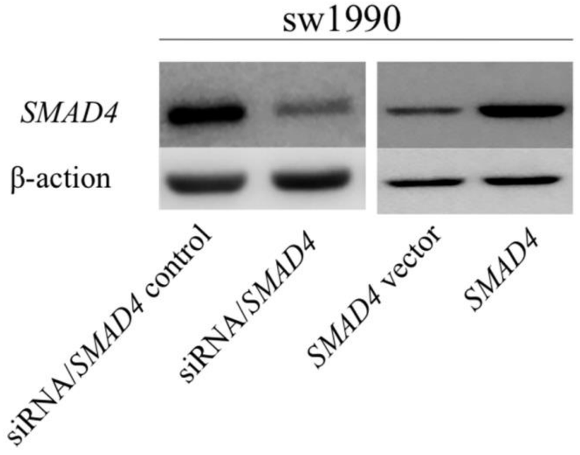

Figure S2: *SMAD4* gene expression was examined via Western blot after *SMAD4* overexpression or knockdown in sw1990 cell.

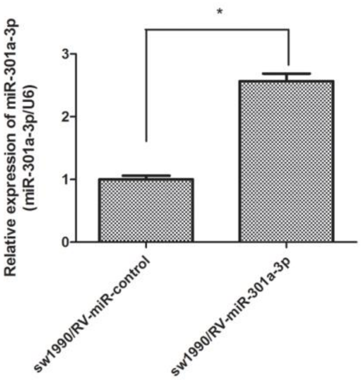

Figure S3: To validate the transfection efficiency of miR-301a-3p in retrovirus-mediated sw1990/ miR-301a-3p and sw1990/ miR-control stable cell lines, qRT-PCR was utilized to measure their miR-301a-3p expression. U6 was used for normalization. Data are shown as mean ± SEM of three independent experiments. *$P < 0.05$。

Table S1: Association between miR-301a-3p ISH score level and PDAC samples in the tissue microarray(n=90)

Tissue microassay	miR-301a-3p expression		P value
Region	High expression	Low expression	0.05
Tumor	46	44	
Corresponding non-tumor	32	58	

Table S2 Association between *SMAD4* IHC score level and PDAC samples in the tissue microarray(n=90)

Tissue microassay	*SMAD4* expression		P value
Region	High expression	Low expression	0.008
Tumor	24	66	
Corresponding non-tumor	41	49	
